# *Primulina serrulata* (Gesneriaceae), a new species from southeastern Guizhou, China

**DOI:** 10.3897/phytokeys.132.36717

**Published:** 2019-09-19

**Authors:** Hong Jiang, Tan Deng, Xin-Yun Lv, Ren-Bo Zhang, Fang Wen

**Affiliations:** 1 Department of Biology, Zunyi Normal College, Zunyi, Guizhou 563002, China Zunyi Normal College Zunyi China; 2 Guangxi Key Laboratory of Plant Conservation and Restoration Ecology in Karst Terrain, Guilin Botanical Garden, Guangxi Institute of Botany, Guangxi Zhuangzu Autonomous Region and Chinese Academy of Sciences, Guilin 541006, China Guangxi Institute of Botany, Guangxi Zhuangzu Autonomous Region and Chinese Academy of Sciences Guilin China; 3 Gesneriad Conservation Center of China, Guilin Botanical Garden, Chinese Academy of Sciences, Guilin 541006, China Guilin Botanical Garden, Chinese Academy of Sciences Guilin China; 4 Key Laboratory of Plant Resources Conservation and Sustainable Utilization, South China Botanical Garden, Chinese Academy of Sciences, Guangzhou 510650, China South China Botanical Garden, Chinese Academy of Sciences Guangzhou China

**Keywords:** Flora of Guizhou, Karst region, New taxa, *Primulina fimbrisepala*

## Abstract

*Primulina serrulata* R.B.Zhang & F. Wen, a new species from a limestone area in southeastern Guizhou, China, is described and illustrated here. The new species is morphologically related to *P. fimbrisepala* (Hand.-Mazz.) Y.Z.Wang. We examined the morphological differences between these congeners and provide illustrations and photographs of this new species in this paper.

## Introduction

The formerly monotypic genus, *Primulina* Hance, has become the largest genus in the subfamily *Didymocarpoideae* of Gesneriaceae in China (Weber et al. 2013, Xu et al. 2017, Wen et al. 2019). The number of *Primulina* has been growing explosively since 2005 (Möller et al. 2016, Möller 2019). For the moment, there are a total of 199 species (including infraspecies) in this genus in China (Wen et al. 2019). *Primulina* shows high levels of endemism and ecological (edaphic) specialization, especially in Karst areas. Based on extensive literature surveys and field observations, we discovered that most species of *Primulina* occur in the karst area of southern and southwestern China and northern Vietnam. They are often limited to a single or a few caves or in specialized and narrow microhabitats of karst limestone hill systems, called island distributions (Wang et al. 1998, Li and Wang 2004, Wei et al. 2010, Hao et al. 2015). The current research result indicated that geographical isolation had been demonstrated to be one kind of reliable driver of *Primulina* diversification and speciation, for example, *P. eburnea* and *P. hochiensis* complexes (Gao et al. 2015, Wang et al. 2017, Yang et al. 2019).

**Figure 1.**
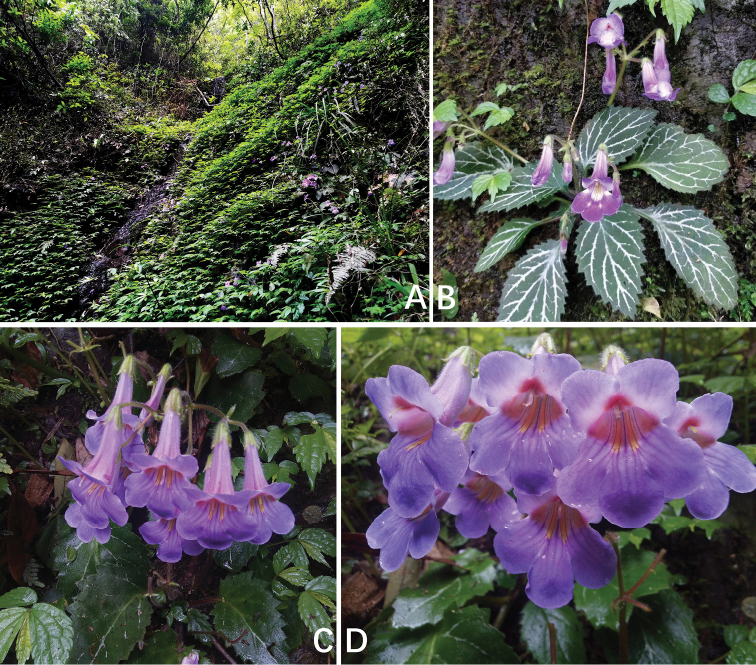
*Primulina serrulata*. **A** natural habitat **B** habit in flowering **C** cyme and flower in top view **D** flowers in front view. Charted by Wen-Hua Xu.

In 2018, during field explorations, a local herbalist found an unknown species of *Primulina* near the Guizhou-Guangxi border at Rongjiang County, southeastern Guizhou, China (Figure 1). Several living individuals from the population found in the field were brought to the nursery of Gesneriad Conservation Center of China (GCCC) and cultivated there. The leaf blade margin characteristics of these plants look appealing because of the prominent and irregular serrations. At the same time, the beautiful silvery veins on the leaf blades and purplish-blue flowers soon caught our attention. However, this species can easily be distinguished from its congener by its morphology. After a morphological comparison between this new species and its related species and literature studies, we consider it is new to science, which is being described and illustrated here.

## Methods

Morphological observations of the new species were carried out based on living plants as well as dry specimens. All morphological characters were measured with dissecting microscopes and were described using the terminology presented by Wang et al. (1998). Literature studies included all relevant monographs, i.e., Wang et al. (1998), Li and Wang (2004) and Wei et al. (2010), and also some recently published literature. Specimen checking was done at IBK, IBSC, PE and also some web databases, such as Chinese Virtual Herbarium (http://www.cvh.ac.cn/) and Global Plants (http://plants.jstor.org/).

## Taxonomy

### Primulina serrulata

Taxon classificationPlantaeLamialesGesneriaceae

R.B.Zhang & F.Wen sp. nov.

A3644B0E-FA5E-5902-A8EC-38C4109E6739

urn:lsid:ipni.org:names:77201948-1

Figures 1 , 2 , 3

#### Diagnosis.

*Primulina serrulata* mainly differs from its congener, *P. fimbrisepala*, by its purplish-blue flowers which lack the dark purple spots inside the corolla (*vs.* purple to purplish-pink, with bright dark purple spots inside the corolla), cuneate leaf blade base (*vs.* obliquely cuneate, broadly cuneate or cordate), anthers glabrescent (*vs.* sparsely bearded) and smaller stigma ca. 1 mm long (*vs.* 2–3 mm long).

#### Type.

CHINA. Guizhou Province, Rongjiang County, Langdong town, growing on moist limestone rocks surfaces near waterfall, alt. 780 m, 26°07'N, 108°42'E, 17 April 2018 (flowering), *Ren-Bo Zhang et al.*, *ZRB1478* (holotype: ZY!; isotype: IBK!).

**Figure 2.**
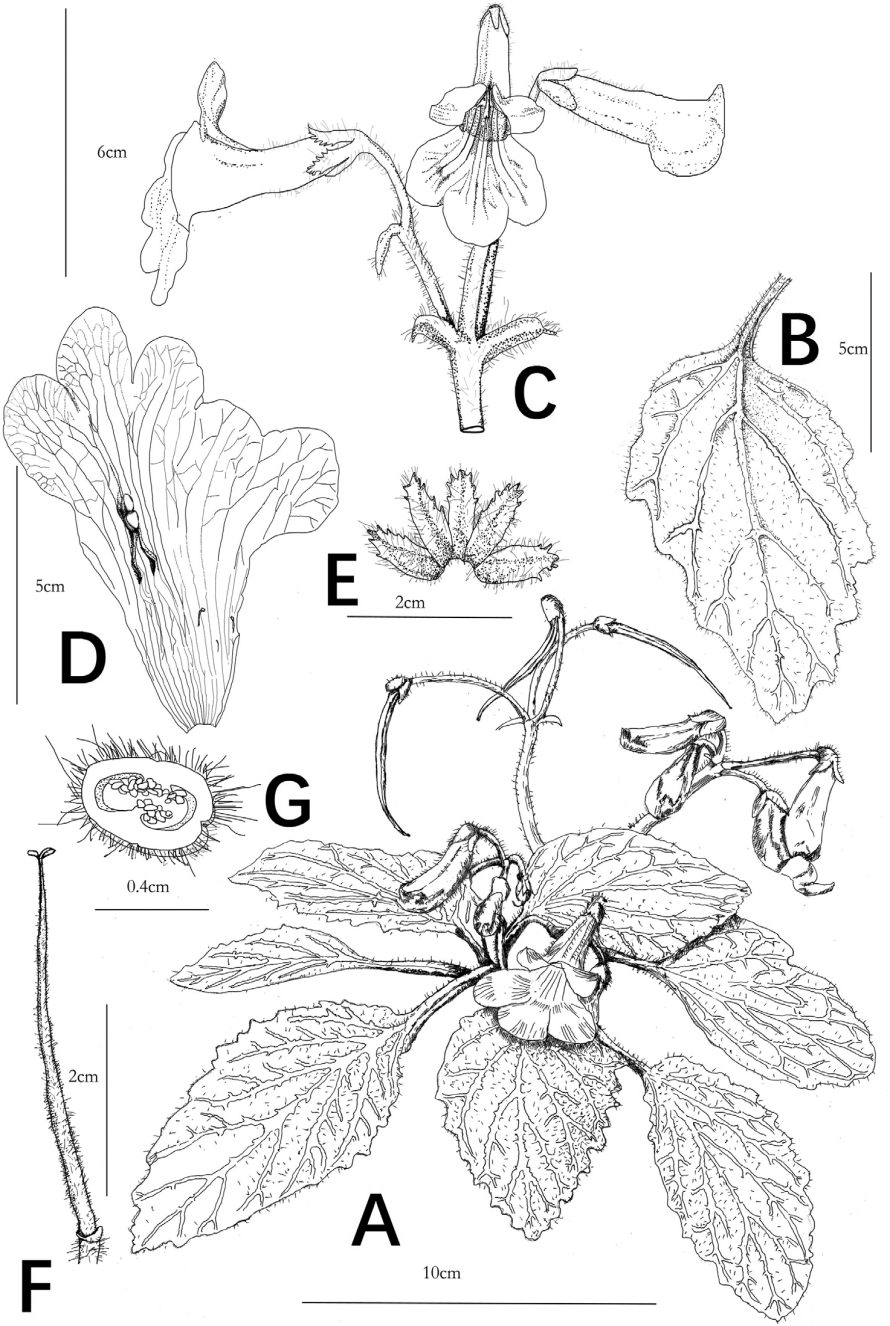
*Primulina serrulata*. **A** habit **B** adaxial leaf blade **C** cyme, bracts, flower in frontal view and flower in lateral view **D** opened corolla for showing stamens and staminodes **E** adaxial calyx lobes **F** pistil **G** cross section of ovary. Drawn by Tan Deng.

#### Description.

***Rhizomatous*** stem subterete, ca. 6 cm long, ca. 1.5 cm in diameter. **Leaves** 4–6, all basal, opposite decussate. ***Petioles*** flatted, fragile and easy to be broken, 2–7 cm long, 4–5 mm wide, densely erect white multicellular hispid, hairs ca. 7 mm long. ***Leaf blade*** slightly fleshy and fragile when fresh, chartaceous when dried, obliquely ovate, oval to nearly rounded, 5–13 × 3–10 cm, densely erect white multicellular hispid and short hispid on both surfaces, base obliquely broadly cuneate, cordate to nearly rounded, margin conspicuously big and irregular serrate and biserrate; ***lateral veins*** 3–5 on each side, adaxially inconspicuously sunk but clearly slivery, occasionally green, abaxially conspicuously raised. ***Cymes*** 1–6, axillary, 1–2 branched, 2–5(8)-flowered; ***peduncles*** 11–16 cm long, ca. 2 mm in diameter, densely erect white multicellular hispid and short hispid; ***bracts*** 2, green, opposite, narrowly rhombic, 5–12 × 2–5 mm, margin inconspicuously serrate, apex acute, outside densely pubescent, inside pubescent; sometimes with bracteoles, opposite, 2, narrowly triangle, ca. 5 × 1 mm. ***Pedicel*** 2.5–5.5 cm long, ca. 1 mm in diameter, densely pubescent. ***Calyx*** 5-parted to near base, lobes oblong to lanceolate, green, 8–15 × 3–5 mm, outside densely pubescent, inside glabrous, apex subacute, margin entire about 2/3 of calyx lobe from the base but denticulate 3–4(5) about 1/3 of calyx lobe from the apex. ***Corolla*** pale purple to purple, the color of the throat same as corolla with two longitudinal yellow stripes along corolla tube but without dark purple spots, 4.5–6.0 cm long, outside glandular-pubescent and puberulent from the base to middle of corolla tube, inside glabrous; ***tube*** infundibuliform-tubular, 3.0–3.5 cm long, 1.0–1.5 cm in diameter at mouth, ca. 4 mm in diameter at base; ***limb*** distinctly 2-lipped, adaxial lip 2-parted, the upper part of the interior of two adaxial lip lobes with two lines of glandular hairs on the brown patch, lobes broadly ovate, 8–12 × 5–9 mm, apex rounded, abaxial lip 3-lobed, lobes oblong, 2.0–2.8 × 1.0–1.4 cm, apex rounded. ***Stamens*** 2, adnate to 10–13 mm above the corolla tube base; ***filaments*** linear, ca. 15 mm long, white, geniculate near the base, sparely puberulent from the middle to the top, the rest glabrous; ***anthers*** fused by the entire adaxial surfaces, ca. 4 mm long, glabrous. ***Staminodes*** 3, lateral ones 6–7 mm long, adnate to 12–15 mm above the corolla tube base, middle one ca. 1.5 mm long, adnate to 8–10 mm above the corolla tube base. ***Disc*** annular, the higher side ca. 1.5 mm in height but the lower side ca.0.8 mm in height. ***Pistil*** 3.5–4.5 cm long; ***ovary*** cylindrical, 2.5–3.2 cm long, densely glandular pubescent and puberulent; ***style*** 1–1.3 cm long, densely glandular-pubescent and puberulent; ***stigma*** obtrapezoid, apex 2-lobed, ca. 1 mm long, ca. 0.8 mm wide. ***Capsule*** linear, ca. 6 cm long, sparsely pubescent.

**Figure 3.**
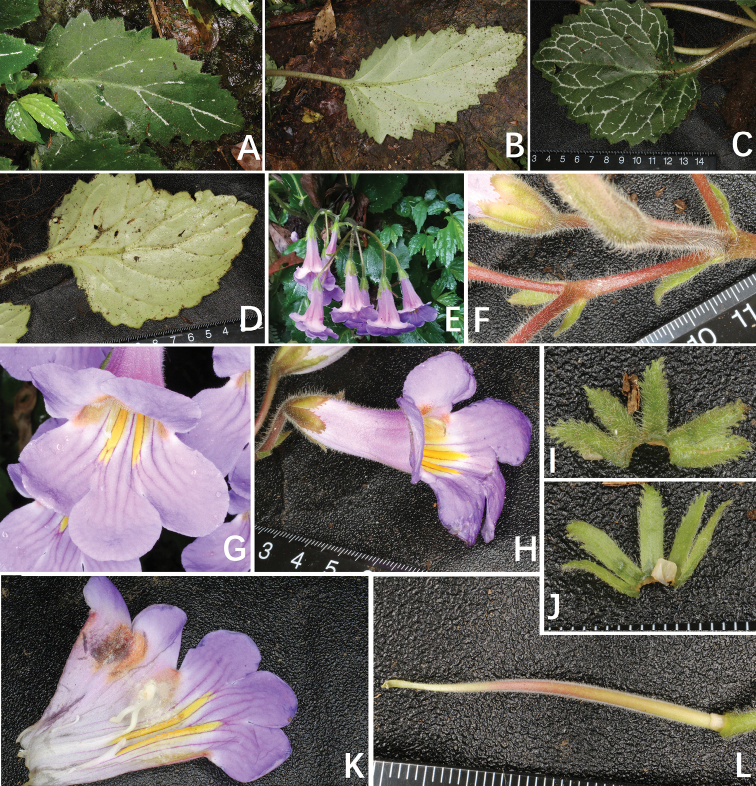
*Primulina serrulata*. **A, C** different shapes of adaxial leaf blades **B, D** different shapes of abaxial leaf blades **E** cyme, bracts, flower in top view **F** bracts and bracteoles **G** corolla in frontal view **H** corolla in lateral view **I** adaxial calyx lobes **J** abaxial calyx lobes **K** opened corolla **L** pistil. Charted by Wen-Hua Xu.

#### Distribution and habitat.

At this time, *Primulina serrulata* is only known from the type locality in Langdong village, Langdong town, Rongjiang County, Guizhou Province, based on our field investigations. It grows on moist, shady, limestone rocks near a waterfall, at ca. 780 m altitude, with no more than 150 mature individuals. The population is close to a road, which makes it vulnerable and subject to destruction from human activities.

#### Phenology.

This new species was observed flowering in April and fruiting from May to June.

#### Etymology.

The specific epithet is derived from its particular leaf blade margin, having obvious serrations and bi-serrations.

#### Provisional IUCN conservation assessment.

Because of *Primulina serrulata*’s beautiful leaves and flowers, it is being over-harvested by local people for sale. This unpublished species is therefore on the brink of extinction as a result. Before more surveys are completed to clarify its conservation status, the provisional conservation status is Critically Endangered CR B2ab (iii, v) according to the IUCN red list criteria (IUCN 2012).

#### Note.

**Figure 4.**
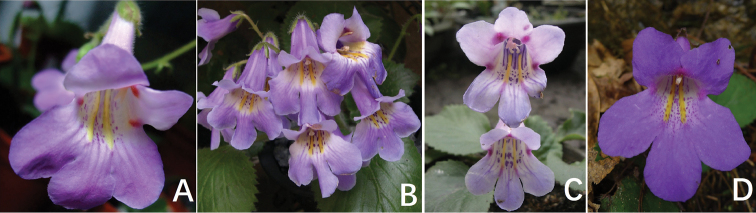
The corolla throat character of *Primulina serrulata*’s congener, *P. fimbrisepala*. **A** from Wenzhou city, Zhejiang **B** from Ziyuan County, Guangxi **C** from Jinxiu County, Guangxi **D** from Ruyuan County, Guangdong. Photoed by Fang Wen, Charted by Wen-Hua Xu.

*Primulina serrulata* is related to its congener, *P. fimbrisepala*, by some characteristics, for example, the similar calyx lobes and infundibuliform corolla tube, but they can easily be distinguished from each other by the characters summarized in the description below. Numerous dark purple spots were covered at the throat of the corolla, and this is one stably distinctive feature of *P. fimbrisepala*. It is noticeable in different populations of *P. fimbrisepala* from South China when they are flowering (Figure 4). Table 1 below has more detailed information on how to distinguish the two species. They also grow in different substrates. *P. serrulata* only grows in limestone areas, while *P. fimbrisepala* commonly appears in weakly acidic mediums and soils of sandstone or granite mountainous regions. The different growing habitats of the two congeners indicate that geographical isolation should be one of the reliable drivers pushing this genus, *Primulina*, to generate diversification and speciation (Gao et al. 2015, Kong et al. 2017, Wang et al. 2017, Yang et al. 2019).

**Table 1.** Morphological comparison of *Primulina serrulata* sp. nov. and *P. fimbrisepala*.

Characters	*P. serrulata*	*P. fimbrisepala*
Indumentum of leaf blade	densely erect white multicellular hispid and short hispid on both surfaces	adaxially puberulent and appressed pilose, abaxially sparsely puberulent to velutinous
Indumentum of peduncles	densely erect white multicellular hispid and short hispid	pubescent to appressed pilose
Calyx lobes size	8–15 × 3–5 mm wide	7–11 × 1.5–3.0 mm wide
Corolla color	pale purple to purple, throat without dark purple spots	blue, purple, to pinkish green, throat with numerous dark purple spots
Filaments length	ca. 1.5 cm long	ca. 1.3 cm long
Indumentum of anthers	Glabrescent	sparsely bearded
Stigma size	ca. 1 mm long, ca. 0.8 mm wide	2–3 mm long, ca. 1 mm wide
Flowering time	late April to early May	March to early April

## Supplementary Material

XML Treatment for Primulina serrulata
